# Systematic Review of Nutrition Supplements in Chronic Kidney Diseases: A GRADE Approach

**DOI:** 10.3390/nu13020469

**Published:** 2021-01-30

**Authors:** Pei-Chin Lin, Chu-Lin Chou, Shih-Hsiang Ou, Te-Chao Fang, Jin-Shuen Chen

**Affiliations:** 1 Department of Medical Education and Research, Kaohsiung Veterans General Hospital, Kaohsiung 813414, Taiwan; pclin@vghks.gov.tw; 2 Department of Pharmacy, School of Pharmacy, Kaohsiung Medical University, Kaohsiung 807017, Taiwan; 3 Division of Nephrology, Department of Internal Medicine, School of Medicine, College of Medicine, Taipei Medical University, Taipei 110301, Taiwan; chulin.chou@gmail.com; 4 Division of Nephrology, Department of Internal Medicine, Shuang Ho Hospital, Taipei Medical University, New Taipei 235041, Taiwan; 5 TMU Research Center of Urology and Kidney, Taipei Medical University, Taipei 110301, Taiwan; 6 Division of Nephrology, Department of Internal Medicine, Kaohsiung Veterans General Hospital, Kaohsiung 813414, Taiwan; shou@vghks.gov.tw; 7 Division of Nephrology, Department of Internal Medicine, Taipei Medical University Hospital, Taipei Medical University, Taipei 110301, Taiwan; 8 National Defense Medical Center, School of Medicine, Taipei 114201, Taiwan

**Keywords:** chronic kidney disease, vitamin D and analogues, omega-3 polyunsaturated fatty acid, dietary fiber, coenzyme Q10, biotics

## Abstract

Chronic kidney disease (CKD) is cumulative worldwide and an increasing public health issue. Aside from the widely known protein restriction and medical therapy, less evident is the renal protection of nutrition supplements in CKD patients. This systematic review (SR), using a Grading of Recommendations Assessment, Development, and Evaluation (GRADE) approach, aims to summarize and quantify evidence about the prevention effects of vitamin D and analogues, omega-3 polyunsaturated fatty acid (omega-3 PUFA), dietary fiber, coenzyme Q10 (CoQ10), and biotics on CKD progression. This study was conducted following the Preferred Reporting Items for Systematic Reviews and Meta-Analyses (PRISMA) statement to examine SRs and/or meta-analysis of clinical controlled trials identified from PubMed, Embase, and the Cochrane Library. Finally, seventeen SRs were included in the qualitative analysis. The beneficial effects of these nutrition supplements in CKD patients mostly seem to be at low to very low evidence on proteinuria, kidney function, and inflammations and did not appear to improve CKD prognosis. The recommendation of nutrition supplements in CKD patients needs to discuss with physicians and consider the benefits over the adverse effects. Longer follow-up of larger randomized trials is necessary to clarify the benefits of nutrition supplements in CKD patients.

## 1. Introduction

Chronic kidney disease (CKD), a gradual loss of kidney function, is an increasing public health issue. Over the past decades, CKD incidence is cumulative worldwide [1,2,3], paralleling epidemics in diabetes [4], hypertension [5], and metabolic syndrome [6]. Prevalence is estimated to be 8–16% worldwide [7]. Complications of CKD are associated with the risk of all-cause mortality, cardiovascular events, hospitalization, cognitive decline, and fracture [8,9,10,11]. Recently, some prognostic biomarkers are developed and helped to improve risk stratification and anticipate diagnosis of cardiovascular diseases [12]. However, CKD prognosis that is ameliorated by patient awareness and management strategies could be considered as an essential issue.

Dietary management to stop CKD progress has some benefits in patients with heavy proteinuria from some large trials [13,14], which were consistent with some reports from smaller-population trials [15,16,17]. The benefits of dietary protein restriction need to subsequently evaluate the problems of malnutrition and protein wasting syndrome [13,14]. Awareness of the disorder and strategies to reduce medical costs related to CKD need to be included in public policy and receiving increasing attention.

Aside from the widely known protein restriction and medical therapy, nutrition supplements have been reported to have a role in CKD prevention [18,19,20,21,22,23,24,25]. For example, a meta-analysis providing data for 688 patients has displayed that active vitamin D analogs reduced proteinuria (−16% (95% confidence interval (CI), −13% to −18%)) compared with controls (+6% (95% CI, 0% to +12%); *p* < 0.001) [18]. Additionally, the meta-analysis in diabetes patients indicated that vitamin D analogs provide beneficial effects on proteinuria and inflammation indexes [high-sensitivity C-reactive protein (hs-CRP), interleukin 6 (IL-6) or tumor necrosis factor-alpha (TNF-α)], but not on serum creatinine and estimated glomerular filtration rate (eGFR) [21]. However, other studies on meta-analysis have the contrary results of proteinuria protection [26,27]. Thus, the effects of vitamin D analogs on kidney disease merit to investigate and clarify in CKD patients.

In addition to vitamin D analogs, lower dietary acid loads (e.g., more fruits and vegetables and fewer meats and cheeses) had been reported to prevent CKD progress in clinical studies [19,20,24]. Other nutrition supplements [e.g., Omega-3 polyunsaturated fatty (Omega-3 PUFA), and biotics] had been reported to help maybe improve kidney function [22,23,25]. These nutritional supplements may have been used in a clinical application until now.

The SRs and/or meta-analysis are the level I study designs to answer the intervention question in clinical practice. Nevertheless, the previous SRs on the relationship between nutrition supplements and CKD were inconsistent and the evidence certainty was lacking. The Grading of Recommendations Assessment, Development, and Evaluation (GRADE) approach offers a transparent and structured process for developing and presenting summaries of evidence, including its effect size and certainty, and recommendations in health care. The objective of this systematic review was to determine the effects of current and relevant SRs of nutrition supplements on renal protection in CKD patients through using the GRADE approach.

## 2. Materials and Methods

### 2.1. Study Methods

This systematic reviews (SRs) was conducted and reported following the Preferred Reporting Items for SRs and Meta-Analyses (PRISMA) statement [28] to examine the renal protection effect of nutrition supplements in CKD (Table S1).

### 2.2. Search Strategy

We searched PubMed, Embase, and Cochrane CENTRAL databases for relevant SRs from the inception through October 2020. The strategy and keywords used for the systematic search were (“herbal supplements” OR “herbal supplement” OR “dietary supplements” OR “dietary supplement” OR “nutritional supplements” OR “nutritional supplement” OR supplementation* OR “nutrition supplements” OR “nutrition supplement” OR “health foods” OR “health food”) AND (renal or kidney). We applied a high-sensitivity and high-specificity customized filter [29], a sensitivity of 96.0% and a specificity of 99.96%, for efficiently retrieving SRs. We also performed hand searches from the relevant studies of the included SRs to identify additional studies.

### 2.3. Selection Criteria

Inclusion criteria included: (i) study design: SRs and/or meta-analysis of clinically controlled trials (whether randomization was applied will be judged in quality assessment) designed to evaluate the influence of nutrition supplementation on renal protection in CKD patients compared to placebo, regardless of language or publish date, (ii) population: any stages of CKD patients should not need dialysis or renal transplantation at baseline, (iii) intervention: nutrition or health supplements, (iv) comparison: placebo or other treatment, (v) renal protection outcomes: a study reported at least one clinical important issue or surrogate outcome data or outcome data can be extracted from subgroup analysis was available. The critical clinical outcomes included kidney function change from baseline and the risk of progression to end-stage renal disease (ESRD); the surrogate outcomes included inflammatory factors, for instances, hs-CRP, CRP, indoxyl sulphate (IS), or p-cresyl sulphate (PCS), and oxidative stress marker, malondialdehyde (MDA). Exclusion criteria: (i) nutrition supplement that has obtained FDA approval of drug license for CKD patients, (ii) Chinese herbal medicine, (iii) studies were presented as conference abstracts, case reports, letters to editors, or in vitro studies.

### 2.4. Data Extraction

A piloted form of data collection was created in excel, and two authors (Lin, Chou) extracted data from the included SRs independently. Collected variables included the first author, publish year, search databases, search duration, included study design and numbers, critical appraisal tool, population, intervention (dose and frequency if available), control group, outcomes related to kidney function, and nutrition supplement duration. Data were collected from publishing papers or online supplements, from study groups or subgroups. If the author provided the original data of included controlled trials and only used the text to describe the significance of effect size rather than presented the statistical data, we further calculated the effect size by author-reported statistical methods. Similarly, if the author only describes overall appraisal results rather than the present individual outcomes-related risk of bias, we further appraise the quality of the subgroup based on the data provided in the SR. Any discrepancies on data extraction or quality assessment were discussed and reached an agreement by consulting with the third author (Chen).

### 2.5. Grading of Recommendations Assessment, Development and Evaluation (GRADE) Grading the Evidence

We rate the certainty of included SRs using the GRADE approach as adopted for the “summary of findings” table. GRADE specified the quality of evidence to four categories—high, moderate, low, and very low. If the context of an SR comes from randomized-controlled trials, the evidence category begins as high-quality. There are five reasons to possibly rate down 1–2 grade of evidence, including the risk of bias (most information comes from studies at moderate or high risk of bias), imprecision (the sample size or “optimal information size” [OIS] criterion is not met or the 95% CI overlap no effect), inconsistency (substantial heterogeneity between studies and unexplained, *I^2^* more than 50% was set as substantial heterogeneity) [30], indirectness (depend on the extent of differences between our interests and the SR on patient populations, interventions, measurements of the outcome, and the methods of the trials of the candidate interventions) and publication bias (asymmetric funnel plots presented, Deeks’ test or the trim and fill method of non-significance, or included studies come from several small studies and most of which were commercially funded) [31,32,33,34,35,36,37].

### 2.6. Data Synthesis and Statistics

Since the included SRs, aim to evaluate the renal protection effect on the same nutrition supplement, may consist of duplicated RCTs and thus become independent, we abandoned to summarize the data by a statistical method and present the result as an SR. If standardized mean difference (SMD) was used in an SR to show the pooled outcome measure; we considered a SMD of 0.2, 0.5, and 0.8 a small, moderate, and large effect, respectively [38]. We used GRADEpro GDP software (GRADEpro GDT: GRADEpro Guideline Development Tool (Software). McMaster University, 2020 (developed by Evidence Prime, Inc.: Hamilton, ON, Canada). Available from gradepro.org.) to synthesis and present the certainty and summary of findings for the included SRs.

## 3. Results

### 3.1. Baseline Information of Included SRs

**Figure 1:**
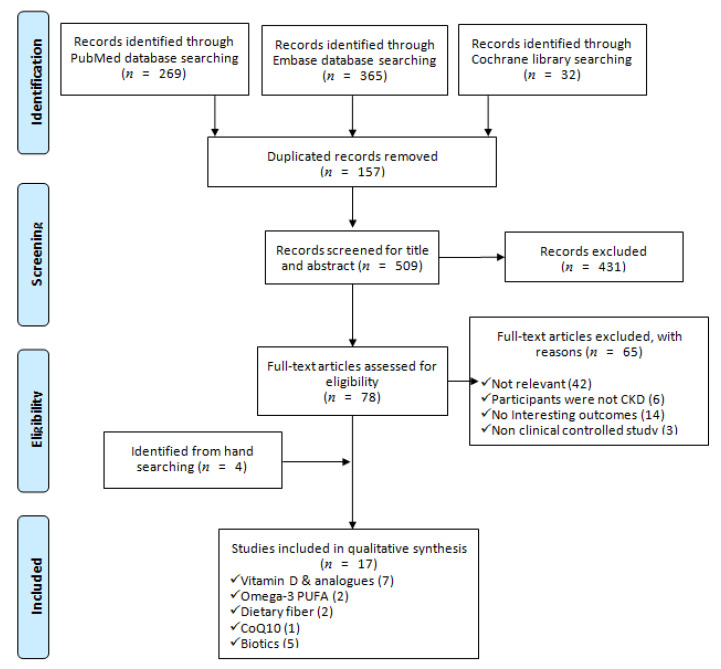
PRISMA flow diagram of including systematic reviews in this study [28].

We identified 666 articles from the electronic databases searching, excluding duplicates, irrelevant, and not fulfilled the inclusion criteria and left 13 SRs. Furthermore, we retrieved 4 SRs by hand searching from the reference lists of relevant studies, eventually included 17 SRs in this SR. Figure 1 outlined the process of systematic search and adding a PRISMA flow diagram. PRISMA checklist was provided as supplementary material (Table S1).

Among the included SRs, 7, 2, 2, 1 and 5 studies aim to evaluate the renal protective effects of vitamin D and analogs [21,26,27,39,40,41,42], Omega-3 PUFA [22,43], dietary fiber [24,44], coenzyme Q10 (CoQ10) [45], and biotics [23,25,46,47,48], respectively. All of the included SRs performed the search on at least three databases, and the majority of searching, except for Zhang et al. study [48], had no restriction on publication date. Five SRs evaluated the effects of nutrition supplements in diabetic nephropathy patients only, and the others in any stages and any etiologies of CKDs. The most used appraisal tools for included RCRs was a Cochrane risk of bias (RoB), 10 SRs were using this checklist. Additionally, there were 2, 1 and 1 studies using the Jadad Scale, Newcastle-Ottawa Scale, and Heyland Methodological Quality Score to appraise the quality of included RCTs, respectively. Moreover, one study assessed the quality by RoB and GRADE simultaneously. In the SRs of vitamin D and analogues, 4 assessed the effects of established vitamin D compounds (vitamin D2, eregocalcifefol, ercalcidiol, ercalcitriol, vitamin D3, cholecalciferol, carcidiol, calcitriol) or the newer analogues (paricalcitol and doxercalcigerol) [26,40,41,42], the others only focused on established compounds. In the biotics SRs, 2 focused on the effect of probiotics [46,47], 1 on probiotics or prebiotics [25], and 2 on probiotics, prebiotics or synbiotics [23,48]. The included clinically controlled trials in all SRs received nutrition supplements at least 1 week. All detailed information of the included SRs was presented in Table 1.

### 3.2. Grading of Recommendations Assessment, Development and Evaluation (GRADE) Qualifying the Evidence

Only four clinical important outcomes (urinary albumin excretion rate (UAER), reduced proteinuria, the occurrence of ESRD, serum urea) and one surrogate outcome (CRP) reported in the five individual SRs [27,40,43,47,48] reached the moderate level of certainty, the other evidence rated low to very low (Table 2 and Table 3). The most being downgraded domain was the risk of bias in included RCTs. In this SR, several outcome data were extracted from subgroup analysis in the original SR. Although the overall quality of the primary outcome may be presented as low or unclear risk of bias, the other outcome-specific risks of bias were not assessed and reported. Thus, downgrading one point was performed. Furthermore, nine clinical relevant outcome evidence presented a high risk of bias in more than half of RCTs; we downgraded two points to very severe in the domain of risk of bias. In the domain of indirectness, we downgraded all surrogate outcomes one point to severe; on the contrary, the clinically relevant outcomes were not treated the same.

### 3.3. Vitamin D and Analogues on Renal Protection

Five SRs focused on diabetic nephropathy reported 13 clinical relevant outcome data. In very low to low certainty evidence of vitamin D and analogues (established vitamin D compounds) appeared to reduce urinary albumin excretion rate (UAER) (2 SRs, included two and eight RCTs, respectively, mean difference −0.39, 95% CI −0.71 to −0.07 and −67.36, 95% CI −91.96 to −42.76). The vitamin D and analogue effect on urine albumin creatinine ratio (UACR) varied in the included SRs. Very low to moderate certainty evidence showed no significant impact on SRs only enrolled supplement with established vitamin D compounds. SRs included RCTs with receiving newer vitamin D analogue (paricalcitol or doxercalciferol) rather than established compounds alone suggested a significant decrease in UACR (two SRs, included eight and four RCTs, respectively, mean difference −0.49, 95% CI −0.9 to −0.08 and SMD −0.29, 95% CI −0.48 to −0.1). However, the effect seems small, and the evidence was very low. In very low to low certainty of evidence suggested vitamin D and analogues reduce proteinuria 0.23–0.26 gm per 24 h (2 SRs, included 11 and 14 RCTs, respectively, mean difference −0.26, 95% CI −0.34 to −0.17 and −0.23, 95% CI −0.3 to −0.15). The effect on serum creatinine varied and the certainty of the evidence was very low. Only one SR reported vitamin D and analogues does not affect urine protein creatinine ratio (UPCR) and eGFR, respectively (Table 2 and Table 4).

Two SRs focused on diabetic nephropathy reported four surrogate outcome data. Very low certainty of evidence suggested Vitamin D and analogues decreased the inflammatory markers of hs-CRP, IL-6, and TNF-α (hs-CRP: MD −0.80 to −0.69; IL-6: −0.73; TNF-α: −56.79), as shown in Table 3.

One SR of vitamin D analogues, as compared with placebo, was investigated on renal protection in CKD patients. Moderate certainty evidence showed significantly reduced proteinuria (relative risk of reduced proteinuria 2.0, 95% CI 1.42 to 2.81) (Table 2 and Table 4).

### 3.4. Omega-3 Polyunsaturated Fatty Acid (PUFA) on Renal Protection

Table 5 showed the effects of omega-3 PUFA on eGFR, progression to ESRD, serum creatinine (SCr), proteinuria and creatinine clearance (CCr) from 2 SRs. In very low to moderate evidence appeared to reduce progression to ESRD significantly and consistently (relative risk, 0.3, 95% CI 0.09 to 0.98 and 0.49, 95% CI 0.24 to 0.99). The effect on reducing proteinuria varied, and the certainty of evidence was low (Table 5).

### 3.5. Dietary Fiber on Renal Protection

One SR reported two clinical relevant outcome data. Very low certainty evidence suggested that dietary fiber has no effect on Scr or serum urea (Table 2). One SR reported two surrogate outcome data comparing dietary fiber to placebo. Low certainty evidence suggested dietary fiber decreased uremic toxin PCS significantly (1 SR, 7 RCTs, MD –16.16, 95% CI −23.824 to −8.492), but not indoxyl sulphate (IS) (Table 3).

### 3.6. Coenzyme Q10 (CoQ10) on Renal Protection

Very low certainty of evidence suggested CoQ10 decreased MDA significantly (1 SR, 2 RCTs, SMD –1.29 SD, 95% CI −2.32 to −0.26) (Table 3), but no effect on clinically important outcomes, SCr and serum urea (Table 2).

### 3.7. Probiotics, Prebiotics and Synbiotics on Renal Protection

Table 6 shows the effect of biotics on serum urea, BUN, and SCr. Three SRs suggested biotics effects on serum urea varied; however, significant decrease serum urea compared to placebo (low certainty: MD −2.12 mmol/L, 95% CI −3.86 to −0.37; moderate certainty: MD −5.0 mmol/L, 95% CI −9.45 to −0.54) with low to moderate certainty evidence. Very low certainty of evidence suggested biotics did not affect BUN and SCr.

Low to moderate certainty evidence suggested biotics decreased MDA, CRP and PCS significantly (MDA: 1 SR, 4 RCTs, SMD –0.79 SD, 95% CI −1.38 to −0.20; CRP: 1 SR, 3 RCTs, SMD –0.71 SD, 95% CI −1.01 to −0.40; PCS: 1 SR, 2 RCTs, MD −0.70, 95% CI −1.4 to −0.01), but no effect on IL-6 (Table 3).

This section may be divided by subheadings. It should provide a concise and precise description of the experimental results, their interpretation, as well as the experimental conclusions that can be drawn.

## 4. Discussion

SRs through the GRADE approach recommended by the Cochrane Collaboration provides rating and strengthening the quality of evidence and has been increasingly adopted by researchers worldwide. The GRADE approach divides the quality of SRs into four evidence levels. For example, the high evidence is reliable without side effects; the moderate or low evidence is needed to consult physicians; and, the very low evidence is not recommended. In this SR and meta-analysis, we adopted the GRADE approach to evaluate the beneficial effects of nutrition supplements on CKD prevention. Our findings provided that evidence of the beneficial effects of those nutrition supplements in CKD patients mostly seems to be low to very low evidence on proteinuria, kidney function, and inflammations. Thus, it is recommended in CKD patients to consult physicians for the prescription of nutrition supplements.

### 4.1. Findings and Implications of this Systematic Review

Vitamin D, including vitamin D2 and vitamin D3, is available for over-the-counter purchase and responsible for increasing intestinal absorption of calcium and phosphate and multiple biological effects [49]. Vitamin D3 is the type that most experts recommend to be utilized in clinical practice because of the more stable and potent form [50]. A review of observational studies has shown associations between vitamin D deficiency and risk of CKD, cardiovascular diseases, cancer, diabetes, infectious diseases, and death [49,51]. This study pooled the results of seven SRs that addressed the effects of vitamin D and analogues administered to patients with proteinuria and renal dysfunction. Data of two SRs show that vitamin D and analogues supplementation in patients with diabetes could reduce proteinuria but not affect kidney function [21,52]; however, other SR data show the contrary results of proteinuria protection [26,27]. Furthermore, most results from SRs suggested no effects on kidney function after receiving vitamin D and analogues in diabetes patients [21,52] and CKD patients [40]. Moreover, there is very low evidence that vitamin D and analogues supplements maybe decreased the inflammatory markers of hs-CRP, IL-6, and TNF-α. Through GRADE approach, our results showed that the beneficial effects of receiving vitamin D and analogues seem smaller in diabetes patients, and the certainty of the evidence was low to very low on proteinuria and very low on kidney function and inflammations. In CKD patients, the beneficial effects of vitamin D and analogues supplements were moderate evidence of proteinuria and low evidence of kidney function. Thus, consistent evidence showed vitamin D and analogues decreased the risk of proteinuria and/or UAER in diabetic nephropathy and CKD, but the strength of the evidence is mostly limited by the quality of the individual studies. It is recommended to discuss with a physician before deciding to receive vitamin D and analogues.

Omega-3 PUFA, a class of particular fatty acids with many biological functions, has been reported to or used together with diet and exercise to help lower triglyceride levels in the blood [53]. Reviews of clinical and epidemiological studies have shown the beneficial effects of omega-3 PUFAs supplements on a series of illnesses such as coronary artery disease and heart failure [54], stroke [55], metabolic syndrome [56], and neurodegenerative diseases (Parkinson’s and Alzheimer’s diseases) [57]. Apart from these disorders, there have been found lower levels of serum Omega-3 PUFA in patients with advanced CKD compared with the general population probably due to malabsorption, metabolic changes, and Omega-3 PUFA loss during the dialysis process [58,59,60]. Additionally, Omega-3 PUFA deficiency is independently related to cardiovascular disease in advanced CKD patients [61,62]. In CKD patients without receiving dialysis, Omega-3 PUFA intake may lower the risk of developing end-stage kidney disease [22,43] and cardiovascular deaths [22]. The possible causes have been reported from the pleiotropic effects of Omega-3 PUFA, including reducing blood pressure levels, lessening inflammation, and improving endothelial function, and altering platelet function and blood viscosity [63,64,65,66]. Via using GRADE approach, our data showed that the moderate to very low evidence appeared to reduce progression to ESRD (relative risk, 0.3, 95% CI 0.09 to 0.98 and 0.49, 95% CI 0.24 to 0.99). Moreover, the effect on reducing proteinuria varied, and the certainty of the evidence was low. Due to the inconsistency of variable evidence in different studies on the eGFR, SCr, proteinuria, or CCr, the recommendation of Omega-3 PUFA supplements needs to consider the benefits over the adverse effects in CKD patients after discussing with physicians.

Higher dietary fiber and lower dietary acid loads (e.g., more fruits and vegetables and fewer meats and cheeses) have been reported to reduce the risk of CKD progression [19,20,24]. A meta-analysis of 14 trials involving 143 CKD patients showed the association of higher dietary fiber intake and lower serum urea and SCr levels (MD −1.76 mmol/l, 95% CI −3.00 to −0.51 and MD −22.83 mmol/l, 95% CI −42.63 to −3.02, respectively) [24]; however, the certainty of evidence is at the very low level because of risk of bias and imprecision form analysis of the GRADE approach. Another meta-analysis of 203 CKD patients, showed that dietary fiber decreased uremic toxin PCS levels (MD –16.16, 95% CI −23.824 to −8.492) [44], which also was low certainty of evidence due to small sample size and risks from some data bias and indirectness. In this study, the data showed very low certainty evidence that dietary fiber has no effect on Scr or serum urea. Furthermore, low certainty evidence suggested dietary fiber decreased uremic toxin PCS levels significantly, but did not affect IS levels. Thus, limited evidence showed that dietary fiber intake might reduce the uremic toxins such as serum urea, SCr, and PCS levels, but there is not found any effect on clinically important outcomes. The evidence is insufficient to recommend dietary fiber for kidney protection in CKD patients.

CoQ10, first identified in 1940, is mostly found in meat, fish, and whole grains [67]. CoQ10 generate adenosine triphosphate (ATP) energy to cell [67] and is most commonly used for an antioxidant that helps improve cardiovascular diseases [68,69], heart failure [70], diabetes [71], hypercholesterolemia [72], migraine headache [73], and many other conditions related to lower CoQ10 levels. Furthermore, CoQ10 has been reported to play an essential role in blood metabolic profiles in diabetic kidney disease [45], which indicates lower blood sugar, blood lipid, and MDA levels, but no effect on serum urea and SCr. By using the GRADE approach, this study further showed the very low certainty of evidence on blood metabolic profiles and no impact on kidney function. Thus, CoQ10 intake is insufficiently evident to recommend in CKD patients.

Microbiota dysbiosis is closely associated with many diseases related to chronic inflammations, including obesity, diabetes, cardiovascular diseases, non-alcoholic fatty liver disease, and obesity-induced CKD [74,75,76]. So far, the prescribed prebiotics, probiotics, and synbiotics may show an impact on the amelioration of these diseases [77,78]. However, the safety issue on these biotics supplements is most important, due to the trend of the broad use of these biotics under different clinical circumstances. There have been reported in a few situations of bacteremia, sepsis, fungal infection, or endocarditis following biotics intake, especially for some immunocompromised patients, malnutrition, or suffering from cancer [79,80,81]. In this study, we investigated the effect of biotics on serum urea, BUN, and SCr, and some metabolic profiles such as MDA, CRP, and PCS in CKD patients. Four SRs showed biotics effects on serum urea were inconsistent results and at very low to moderate certainty of evidence due to the risk of data bias and imprecision [23,25,46,47]. Additionally, the biotics intake appeared to decrease MDA, CRP, and PCS levels significantly [46,48]. However, biotics intake did not affect SCr levels in CKD patients [23,46], which was at the very low certainty of the evidence. Thus, biotics supplements seemed only to reduce serum urea and some metabolic profiles in CKD patients. We suggest CKD patients discuss with a physician before considering biotics supplements.

### 4.2. Strengths, Limitations and Further Research Needs

In summary, this study was the first and composed of seven SRs of vitamin D and analogues, two SRs of omega-3 PUFA, two SRs of dietary fiber, one SR of CoQ10, and five SRs of biotics for effect on proteinuria, kidney function, and inflammation in CKD patients. According to the GRADE approach assessment, the strength of evidence was low to very low levels for the benefits of these nutrition supplements on proteinuria and kidney function and did not appear to improve CKD prognosis. The recommendation of these nutrition supplements in CKD patients needs to discuss with physicians and consider the benefits over the adverse effects. Multicenter and large samples of RCTs with more than ten years of follow-up merit to be conducted in the future and to verify the benefits of nutrition supplements in CKD patients.

## Figures and Tables

**Table 1:** Baseline information of included systematic reviews.

Author Year	Search Databases	Search Duration (Through)	Included Study Design	Study No.	Critical Appraise Tool	Population	Intervention	Control	Outcomes Related to Kidney Function	Treatment Duration
**Vitamin D and Analogues**										
Gupta 2019 [26]	PubMed, Scopus, and Google scholar	January 2018	RCTs	9 T2DN (7) T1DN (1) NA (1)	RoB	DN	calcitriol 0.25–0.5 mcg QD-BIW or 50,000IU QW paricalcitol 1–2 mcg QD cholecalciferol 50,000IU QW vitamin D3 50,000IU QM	placebo	UACR, UPCR, 24-h urine protein excretion, UAER, SCr,	8–24 weeks
Milajerdi 2019 [39]	EMBASE, Scopus, PubMed, Cochrane Library, and Web of Science	November 2018	clinical trials	17 non-dialysis (6)dialysis (11)	Cochrane RoB	CKD with or without dialysis	Calcitriol 0.03 mc/kg BIW−0.5 mcg/day Vitamin D3 4662–350000IU/week (QD QW Q2W QM) ergocalciferol 50,000 IU QW-QM	placebo	CRP	3 and 52 weeks
Wang 2019 [21]	Pubmed, Embase, Cochrane Library, CNKI, WANGFANG and VIP	September 2007~July 2018	RCTs	20 T2DN (8) T1DN+T2DN (12)	Cochrane RoB	DN	calcitriol 0.14–1 mcg/day (QD, BIW) alfacalcidol 0.25–0.5 mcg/day cholecalciferol 800 IU/day Vitamin D3 50,000 IU/day	placebo or blank treatment	24-h urine protein; UAER; SCr; eGFR; hs-CRP; TNF-α; IL-6	8–24 weeks
Zhang 2017 [42]	PubMed, Embase, Scopus, Index Copernicus, DOAJ, CNKI, and Wanfang	January 2017	RCTs	24 T2DN (17) T1DN (1) NA (6)	Newcastle–Ottawa scale	DN DN (20) DN+Vit.D deficient (4)	alfacalcidol calcitriol cholecalciferol paricalcitol	placebo	24-h proteinuria, UACR, hs-CRP, SCr	6–24 weeks
Derakhshanian 2015 [27]	PubMed, SCOPUS, and Google Scholar	September 2014	RCTs (4), cross-sectional studies (6)	10	RCTs: Jadad score Cross-sectional studies: Newcastle—Ottawa Scale	DN	RCTs cholecalciferol 5600–50,000 IU/week (QD, QW) calcitriol 20 IU/day	placebo	UACR	RCTs 6–24 weeks
Zhao 2014 [41]	Pubmed, Embase, Sinomed, CNKI, Wanfang and clinical trial register centers	1 January 2014	RCTs	20	RoB	DN	Vitamin D3, paricalcitol, cholecalciferol, calcitrio, alfacalcidol	placebo or blank control	Change of 24-h proteinuria from baseline, UACR, urine microalbumin	F/U: 4–48 weeks
Xu 2013 [40]	PubMed, EMBASE and OvidSP	September 2012	RCTs	18	Cochrane risk of bias	CKD without dialysis or renal transplantation	cholecalciferol 7000–75,000 IU/week (QD, QW, QM) calcitriol 0.25–0.5 mcg QD-BIW paricalcitol 0.86–4 mcg/day, QDTIW doxercalcigerol 1 mcg/day alfacalcidol 0.25–1 mcg QD	placebo or no medication	24 hr-urine protein, UACR, eGFR, CCr	study duration: 1–24 months
**Omega-3 polyunsaturated fatty acid (n-3 PUFA)**										
Saglimbene 2020 [22]	MEDLINE, Embase, and CENTRAL	12 January 2018	RCTs or quasi RCTs	60 CKD stage 1–5 (20) dialysis (24) transplant (16)	GRADE	adults and children with CKD across all stages	Fish or n-3 PUFA supplementation (0.4–12 g/day)	placebo, standard care, or any other treatment	ESKD, acute transplant rejection, and allograft loss	treatment and F/U: 1–48 months
Hu 2017 [43]	PubMed, Embase, and Cochrane Library	October 2014	RCTs	9 CKD IgA nephropathy (7) ADPKD (1)	Jadad score	CKD (not on ESRD)	EPA 1.8–10 g/d, fish oil 6 g/day omega-3 capsules 4 g/day PUFAs 3.0 g/day	placebo, ACEI/ARB, low dose EPA and DHA, RASB, corn oil, olive oil	proteinuria, CCR, eGFR, occurrence of ESRD	F/U: 2–76.8 months
**Dietary fiber**										
Wu 2019 [44]	PubMed, Web of Science and Cochrane Library	1 September 2017	clinical controlled trials; RCTs (6) pre-post trials (6)	12 RCTs (6) pre-post trials (6)	Heyland Methodological Quality Score *	stage 3–5 of CKD with or without dialysis	dietary fibre intake 7.5–25 g/day	placebo	IS, PCS	mean 5 weks 2.1–10 weeks
Chiavaroli 2015 [24]	MEDLINE, EMBASE, CINHAL and Cochrane Library	1 September 2014	controlled feeding trials	14 non-dialysis(10) HD (4)	Heyland Methodological Quality Score *	CKD with or without dialysis	dietary fiber intake (median fiber dose 26.9 g/day (range 3.1–50.0 g/day))	non-fiber supplemented diets or low-fiber diets	serum urea, SCr	F/U median 4.5 weeks (range:1.4–20 weeks)
**Coenzyme Q10 (CoQ10)**										
Zhang 2019 [45]	PubMed, Web of Science, Ovid-Medline, ProQuest, Science Direct, Springer link et al. **	June 2018	RCTs (4), experimental studies (4)	8	Cochrane risk of bias	type 1 or 2 diabetic kidney disease	CoQ10 (30–1000 mg/day) in combination with western medicine or CoQ10	western medicine or placebo	eGFR, Serum Urea, BUN, Scr	12 weeks
**Probiotics, Prebiotics, Synbiotics**										
Zheng 2020 [48]	PubMed, Cochrane CENTRAL, and the Web of Science	1 January 2000~15 May 2019	RCTs	13 CKD stages 2–4 (2) HD (7) DN (4)	Cochrane ROB	DN	Probiotics alone or associated with prebiotics (synbiotics)	placebo	CRP	4–12 weeks
McFarlane 2019 [25]	MEDLINE, CINAHL, EMBASE, Cochrane Central Register of Controlled Trials, and International Clinical Trials Register and clinicaltrials.gov	July 2017	RCTs	16 non-dialysis (8)HD (7) PD (1)	Cochrane RoB	adults and children CKD with or without dialysis	prebiotic 2.3–50 g/day probiotic 11 × 10^6^–2 × 10^12^ CFU/day	placebo	eGFR, SCr, proteinuria, serum urea, free and protein-bound concentrations of serum IS and PCS, progression to ESKD	1–24 weeks
Tao 2019 [47]	PubMed, Embaseand Cochrane	September 2018	RCTs	10 non-dialysis (4) HD (5) PD (1)	Cochrane RoB	CKD with or without dialysis	probiotic supplementation 2 × 10^9^–1.8 × 10^11^ CFU/day	placebo	urea, uric acid, CRP, SCr, eGFR	6–24 weeks
Jia 2018 [46]	PubMed, EMBASE and Cochrane Library	31 March 2018	RCTs	8 non-dialysis (4) HD (3) PD (1)	Cochrane RoB, GRADE	CKD with or without dialysis	Probiotics: 4 × 109–1.8 × 1011 CFU/day	placebo	BUN, SCr, CRP, IL-6	6–24 weeks
Pisano 2018 [23]	Ovid-MEDLINE, PubMed and CENTRAL	5 March 2018	RCTs	17 non-dialysis (10) HD (5) PD (1) transplant (1)	Cochrane renal group, risk of bias	CKD or ESKD on chronic renal replacement	prebiotics 20–50 g/day, probiotics 2# tid; 2 x 10^9^–9 × 10^10^ CFU/day synbiotics: 3–6#/day;1 5 gm powder/day; 1.1 × 10^7^ CFU+inulin 2.31 g/day; 15 g powder+2#/day	placebo or standard therapy	CCr, eGFR, SCr, albuminuria, CRP, serum urea, TNF-α; IL-6	4–24 weeks

*: scores equal to or more than 8 is considered of high methodological quality (high MQS). **: 12 databases were searched, PubMed, Web of Science, Ovid-Medline, ProQuest, Science Direct, Springer link, Wiley Library Online, Chinese BioMedical Literature Database (CBM), China National Knowledge Infrastructure (CNKI), Chinese medical Citation Index (CMCI), Chinese Scientific Journal Database (VIP), and Wanfang database. RCTs: randomized controlled trials; RoB: risk of bias; DN: diabetic nephropathy;T2DN: type 2 diabetic nephtopathy; T1DN: type 1 diabetic nephtopathy; NA: not available; UACR: urine albumin creatinine ratio; UPCR: urine protein creatinine ratio; UAER: urinary albumin excretion rate; SCr: serum creatinine; CKD: chronic kidney disease; CRP: C-reactive protein; eGFR: estimated glomerular filtration rate; hs-CRP: high-sensitivity C-reactive protein; TNF-α: tumor necrosis factor-alpha; IL-6: interleukin 6; CCr: creatinine clearance; GRADE: Grading of Recommendations Assessment, Development and Evaluation; ESKD: end-stage kidney disease; ESRD: end-stage renal diseases; IS: Indoxyl sulphate; PCS: p-cresyl sulphate; ESKD: end-stage kidney diseases; CFU: colony-forming unit; BUN: blood urea nitrogen; MDA: malondialdehyde.

**Table 2:** Grading of Recommendations Assessment, Development, and Evaluation (GRADE) approach evidence certainty and summary of findings of the clinical important outcomes.

			Certainty Assessment					Certainty *	Summary of Findings	
Author year	Outcomes	No of Studies	Risk of Bias	Inconsistency	Indirectness	Imprecision	Publication bias	Overall Certainty of Evidence	Relative Effect (95% CI)	Risk Difference with Nutrition Compared to Placebo (95% CI)
**Vitamin D and Analogues**										
Gupta 2019 [26]	UAER	2 RCTs	serious ^a^	serious ^b^	not serious	not serious	none	⨁⨁◯◯ LOW	-	MD **0.39 lower** (0.71 lower to 0.07 lower)
	UACR	5 RCTs	very serious ^c^	not serious	not serious	serious ^d^	none	⨁◯◯◯ VERY LOW	-	MD **0.14 lower** (0.34 lower to 0.06 higher)
	UPCR	1 RCT	serious ^e^	not serious	not serious	serious ^d^	none	⨁⨁◯◯ LOW	-	MD **0.19 lower** (0.9 lower to 0.51 higher)
Wang 2019 [21]	eGFR	4 RCTs	serious ^f^	not serious	not serious	serious ^d^	publication bias strongly suspected ^g^	⨁◯◯◯ VERY LOW	-	MD **2.13 higher** (2.06 lower to 6.32 higher)
	SCr	9 RCTs	serious ^f^	not serious	not serious	serious ^d^	publication bias strongly suspected ^g^	⨁◯◯◯ VERY LOW	-	MD **0.83 lower** (3.67 lower to 2.02 higher)
	Proteinuria (g/24 h)	11 RCTs	serious ^f^	not serious ^h^	not serious	not serious	publication bias strongly suspected ^g^	⨁⨁◯◯ LOW	-	MD **0.26 lower** (0.34 lower to 0.17 lower)
	UAER	8 RCTs	serious ^f^	very serious ^i^	not serious	not serious	publication bias strongly suspected ^g^	⨁◯◯◯ VERY LOW	-	MD **67.36 lower** (91.96 lower to 42.76 lower)
Zhang 2017 [42]	Proteinuria (g/24 h)	14 RCTs	serious ^j^	very serious ^i^	not serious	not serious	none	⨁◯◯◯ VERY LOW	-	MD **0.23 lower** (0.3 lower to 0.15 lower)
	UACR	8 RCTs	serious ^j^	serious ^b^	not serious	not serious	none	⨁⨁◯◯ LOW	-	MD **0.49 lower** (0.9 lower to 0.08 lower)
	SCr	9 RCTs	serious ^j^	serious ^b^	not serious	serious ^d^	none	⨁◯◯◯ VERY LOW		SMD **0.16 SD lower** (0.42 lower to 0.11 higher)
Derakhshanian 2015 [27]	UACR	4 RCTs	not serious	not serious	not serious	serious ^d^	none	⨁⨁⨁◯ MODERATE		MD **17.99 higher** (35.36 lower to 71.33 higher)
Zhao 2014 [41]	Proteinuria (g/24 h)	9 RCTs	very serious ^k^	not serious	not serious	not serious	publication bias strongly suspected ^l^	⨁◯◯◯ VERY LOW	-	MD **0.44 lower** (0.54 lower to 0.34 lower)
	UACR	4 RCTs	very serious ^k^	not serious	not serious	not serious	publication bias strongly suspected ^l^	⨁◯◯◯ VERY LOW	-	SMD **0.29 SD lower** (0.48 lower to 0.1 lower)
Xu 2013 [40]	eGFR	12 RCTs	serious ^f^	not serious	not serious	serious ^d^	none	⨁⨁◯◯ LOW	-	SMD **0.1 SD lower** (0.24 lower to 0.03 higher)
	risk of dialysis initiation	4 RCTs	serious ^f^	not serious	not serious	serious ^d^	none ^m^	⨁⨁◯◯ LOW	**RR 1.48** (0.54 to 4.03)	
	reduced proteinuria	6 RCTs	serious ^f^	not serious	not serious	not serious	none ^m^	⨁⨁⨁◯ MODERATE	**RR 2.00** (1.42 to 2.81)	
**Omega-3 Polyunsaturated Fatty Acid (Omega-3 PUFA)**										
Saglimbene 2020 [22]	GFR	6 RCTs	serious ^a^	not serious	not serious	serious ^d^	none	⨁⨁◯◯ LOW		SMD **0.22 SD higher** (0.03 lower to 0.48 higher)
	progression to ESKD	3 RCTs	serious ^a^	serious ^n^	not serious	serious ^n^	none ^m^	⨁◯◯◯ VERY LOW ^n^	**RR 0.3** (0.09 to 0.98)	
	SCr	7 RCTs	serious	serious ^o^	not serious	serious ^d^	none ^m^	⨁◯◯◯ VERY LOW	-	MD 2.20 higher (17.63 lower to 22.03 higher)
	Proteinuria (g/24 h)	6 RCT	serious	not serious	not serious	serious ^d^	none ^m^	⨁⨁◯◯ LOW	-	MD 0.16 lower (0.48 lower to 0.15 higher)
Hu 2017 [43]	CCr	6 RCTs	serious ^p^	serious ^b^	not serious	serious ^d^	none	⨁◯◯◯ VERY LOW	-	SMD **0.22 SD higher** (0.40 lower to 0.84 higher)
	eGFR	6 RCTs	serious ^p^	not serious	not serious	serious ^d^	none	⨁⨁◯◯ LOW		SMD **0.14 SD higher** (0.13 lower to 0.42 higher)
	the occurrence of end-stage renal disease	3 RCTs	serious ^p^	not serious	not serious	not serious	none ^m^	⨁⨁⨁◯ MODERATE	**RR 0.49** (0.24 to 0.99)	
	Proteinuria (g/24 h)	7 RCTs	very serious ^k^	not serious	not serious	not serious	none	⨁⨁◯◯ LOW	-	SMD **0.31 SD lower** (0.53 lower to 0.10 lower)
**Dietary Fiber**										
Chiavaroli 2015 [24]	SCr	8 RCTs	very serious ^k^	not serious	not serious	serious ^d^	none ^m^	⨁◯◯◯ VERY LOW		MD **21.97 lower** (52.22 lower to 8.28 higher)
	serum urea	9 RCTs	very serious ^k^	serious ^b^	not serious	serious ^d^	none ^m^	⨁◯◯◯ VERY LOW		MD **2.35 lower** (4.78 lower to 0.08 higher)
**Coenzyme Q10 (CoQ10)**										
Zhang 2019 [45]	serum urea	2 RCTs	serious ^q^	very serious ^i^	not serious	serious ^d^	none	⨁◯◯◯ VERY LOW	-	SMD 1.24 **SD lower** (4.04 lower to 1.55 higher)
**Probiotics, Prebiotics, Synbiotics**										
McFarlane 2019 [25]	serum urea	4 RCTs	very serious ^k^	not serious	not serious	not serious	none	⨁⨁◯◯ LOW	-	MD **2.12 lower** (3.86 lower to 0.37 lower)
Tao 2019 [47]	serum urea	2 RCTs	not serious	serious ^b^	not serious	not serious	none	⨁⨁⨁◯ MODERATE	-	MD **30.01 lower** (56.78 lower to 3.25 lower)
Jia 2018 [46]	BUN	1 RCT	very serious ^r^	not serious	not serious	serious ^d^	none	⨁◯◯◯ VERY LOW	-	MD **5.78 lower** (21.42 lower to 9.86 higher)
	SCr	3 RCTs	very serious ^k^	not serious	not serious	serious ^d^	none	⨁◯◯◯ VERY LOW	-	MD **0.10 higher** (0.11 lower to 0.31 higher)
Pisano 2018 [23]	SCr	7 RCTs	very serious ^k^	not serious	not serious	serious ^d^	none	⨁◯◯◯ VERY LOW	-	MD **0.02 lower** (0.09 lower to 0.05 higher)
	serum urea	5 RCTs	very serious ^k^	not serious	not serious	serious ^d^	none	⨁◯◯◯ VERY LOW	-	SMD **0.20 SD lower** (0.41 lower to 0.01 higher)

The bold of the words in the columns of “summary of findings” means a statistical significance. *: ⨁⨁⨁⨁ means the highest level of evidence certainty; if a ◯ was replaced by a ⨁, it means a downgrade of evidence certainty. There were four levels of certainty, high, moderate, low and very low, represented as ⨁⨁⨁⨁, ⨁⨁⨁◯, ⨁⨁◯◯ and ⨁◯◯◯, separately. CI: Confidence interval; MD: Mean difference; SMD: Standardized mean difference; RR: Risk ratio. Explanations: ^a^. attrition bias observed in included studies; ^b^. substantial heterogeneity (*I*^2^ > 50%) and cannot find the source of heterogeneity; ^c^. bias in allocation concealment, blinding process, or outcome measure in majority of included studies; ^d^. the pooled estimate cannot exclude no effect; ^e^: bias occurred in randomization, allocation concealment and blinding process; ^f^: the majority of included studies presented low or unclear risk of bias, but no quality assessment in individual outcomes; ^g^: asymmetric funnel plot suggests publication bias; ^h^: high level of heterogeneity decreased after subgroup analysis, the results remained unchanged; ^i^: substantial heterogeneity (*I*^2^ > 90%) and cannot find the source of heterogeneity; ^j^: Newcastle–Ottawa scale used for RCTs; ^k^: high risk of bias observed in at least half of studies; ^l^: no mention on publication bias, but only include studies published in English or Chinese language; ^m^: inadequate to assess due to smaller sample size; neither funding bias was observed nor restriction was set for language, publishing year or country to maximize the extent of the searches; ^n^: author judgement; ^o^: The *I*^2^ in overall studies is 66%, effect size presented significance. Authors did the subgroup analysis and the *p*-value of interaction test for subgroup is 0.01, but no mention of decreasing heterogeneity by subgroup analysis; ^p^: approach half studies showed risk of bias; ^q^: high risk in domain of blinding of participants and personnel (performance bias); ^r^: high-risk bias observed in reporting bias and other bias, unclear in randomization and blinding domains.

**Table 3:** GRADE approach evidence certainty and summary of findings of the surrogate outcomes.

			Certainty Assessment					Certainty *	Summary of Findings
Author Year	Outcomes	No of Studies	Risk of Bias	Inconsistency	Indirectness	Imprecision	Publication Bias	Overall Certainty of Evidence	Risk Difference with Nutrition Supplements Compared to Placebo (95% CI)
**Vitamin D and Analogues**									
Milajerdi 2019 [39]	CRP or hs-CRP	5 RCTs	serious ^a^	not serious	serious ^b^	serious ^c^	none	⨁◯◯◯ VERY LOW	MD **0.41 lower** (0.41 lower to 0.27 higher)
Wang 2019 [21]	hs-CRP	7 RCTs	serious ^a^	not serious	serious ^b^	not serious	publication bias strongly suspected ^d^	⨁◯◯◯ VERY LOW	MD **0.69 lower** (0.86 lower to 0.53 lower)
	IL-6	3 RCTs	serious ^a^	not serious	serious ^b^	not serious	publication bias strongly suspected ^d^	⨁◯◯◯ VERY LOW	MD **0.73 lower** (1.03 lower to 0.44 lower)
	TNF-α	3 RCTs	serious ^a^	very serious ^e^	serious ^b^	not serious	publication bias strongly suspected ^d^	⨁◯◯◯ VERY LOW	MD **56.79 lower** (77.05 lower to 36.52 lower)
Zhang 2017 [42]	hs-CRP	7 RCTs	serious ^f^	serious ^g^	serious ^b^	not serious	none	⨁◯◯◯ VERY LOW	MD **0.80 lower** (1.26 lower to 0.34 lower)
**Dietary Fiber**									
Wu 2019 [44]	IS	5 RCTs	serious ^h^	not serious ^i^	serious ^b^	serious ^c^	none	⨁◯◯◯ VERY LOW	MD 0.212 **lower** (2.35 lower to 1.926 higher)
	PCS	7 RCTs	serious ^h^	not serious	serious ^b^	not serious	none	⨁⨁◯◯ LOW	MD 16.160 **lower** (23.824 lower to 8.492 lower)
**Coenzyme Q10 (CoQ10)**									
Zhang 2019 [45]	MDA	2 RCTs	serious ^h^	serious ^g^	serious ^b^	not serious	none	⨁◯◯◯ VERY LOW	SMD 1.29 **SD lower** (2.32 lower to 0.26 lower)
**Probiotics, Prebiotics, Synbiotics**									
Zheng 2020 [48]	MDA	4 RCTs	not serious	serious ^g^	serious ^b^	not serious	none	⨁⨁◯◯ LOW	SMD 0.79 **SD lower** (1.38 lower to 0.20 lower
	CRP	3 RCTs	not serious	not serious	serious ^b^	not serious	none	⨁⨁⨁◯ MODERATE	SMD 0.71 **SD lower** (1.01 lower to 0.40 lower
Jia 2018 [46]	IL-6	1 RCT	not serious	not serious	serious ^b^	serious ^c^	none	⨁⨁◯◯ LOW	MD 0.23 **lower** (0.27 lower to 0.73 higher)
	PCS	2 RCTs	not serious	serious ^g^	serious ^b^	not serious	none	⨁⨁◯◯ LOW	MD 0.70 **lower** (1.4 lower to 0.01 lower)

The bold of the words in the columns of “summary of findings” means a statistical significance. *: ⨁⨁⨁⨁ means the highest level of evidence certainty; if a ◯ was replaced by a ⨁, it means a downgrade of evidence certainty. There were four levels of certainty, high, moderate, low and very low, represented as ⨁⨁⨁⨁, ⨁⨁⨁◯, ⨁⨁◯◯ and ⨁◯◯◯, separately. CI: Confidence interval; CRP: C-reactive protein; hs-CRP: high-sensitivity C-reactive protein; MD: Mean difference; IL-6: interleukin 6; TNF-α: tumor necrosis factor-alpha; IS: Indoxyl sulphate; PCS: p-cresyl sulphate; MDA: malondialdehyde; SMD: Standardized mean difference. Explanations: ^a^: no data available or no quality assessment available in individual outcomes; ^b^: surrogate outcomes for assessment renal protection; ^c^: the pooled estimate cannot exclude no effect; ^d^: asymmetric funnel plot suggests publication bias; ^e^: considerable heterogeneity (*I*^2^ > 75%) and cannot find the source of heterogeneity; ^f^: Newcastle–Ottawa scale was used for quality assessment of RCTs; ^g^: substantial heterogeneity (*I*^2^ > 50%) and cannot find the source of heterogeneity; ^h^: bias observed in randomization or blinding domain, but less than half studies high risk of bias observed in at least half studies; ^i^: heterogeneity (*I*^2^ = 0, *p* = 0.98) was calculated by ourselves according to the data provided by the original SR author.

**Table 4:** Summary of clinical important outcomes and certainties on the renal protection effect of vitamin D and analogues compared to placebo.

Author Year/Clinical Important Outcomes	UAER		UACR		UPCR		eGFR		SCr		Proteinuria		Risk of Dialysis	
	Risk Difference (95% CI)	Certainty	Risk Difference (95% CI)	Certainty	Risk Difference (95% CI)	Certainty	Risk Difference (95% CI)	Certainty	Risk Difference (95% CI)	Certainty	Risk Difference (95% CI)	Certainty	Relative Risk (95%CI)	Certainty
**Diabetic Nephropathy**														
Gupta 2019 [26]	**MD −0.39 (−0.71 to −0.07)**	LOW	MD −0.14 (−0.34 to 0.06)	VERY LOW	MD −0.19 (−0.9 to 0.51)	LOW	-	-	-	-	-	-	-	-
Wang 2019 [21]	**MD−67.36 (−91.96 to −42.76)**	VERY LOW	-	-	-	-	MD 2.13 (−2.06 to 6.32)	VERY LOW	MD −0.83 (−3.67 to 2.02)	VERY LOW	**MD −0.26 (−0.34 to** **−0.17)**	LOW	-	-
Zhang 2017 [42]	-	-	**MD −0.49** **(−0.9 to −0.08)**	LOW	-	-	-	-	SMD −0.16 SD (−0.42 to 0.11)	VERY LOW	**MD −0.23 (−0.3 to** **−0.15)**	VERY LOW	-	-
Derakhshanian 2015 [27]	-	-	MD 17.99 (−35.36 to 71.33)	MODERATE	-	-	-	-	-	-	-	-	-	-
Zhao 2014 [41]	-	-	**SMD −0.29 SD** **(−0.48 to −0.1)**	VERY LOW	-	-	-	-	**MD −0.44 (−0.54 to −0.34)**	VERY LOW	-	-	-	-
**Chronic Kidney Disease**														
Xu 2013 [40]	-	-	-	-	-	-	SMD −0.1 SD (−0.24 to 0.03	LOW	-	-	**Reduced proteinuria: RR ^a^ 2.00** **(1.42 to 2.81)**	MODERATE	1.48 (0.54 to 4.03)	LOW

The bold of the words in the context means a statistical significance. ^a^: Relative risk (95%CI); UAER: urinary albumin excretion rate; UACR: urine albumin creatinine ratio; UPCR: urine protein creatinine ratio; eGFR: estimated glomerular filtration rate; SCr: serum creatinine; CI: Confidence interval; MD: Mean difference; SMD: Standardized mean difference; RR: Risk ratio.

**Table 5:** Summary of clinical important outcomes and certainties on the renal protection effect of Omega-3 polyunsaturated fatty acid compared to placebo.

Author Year/Clinical Important Outcomes	eGFR		Progression to ESKD		SCr		Proteinuria		CCr	
	Risk Difference (95% CI)	Certainty	Relative Risk (95% CI)	Certainty	Risk Difference (95% CI)	Certainty	Risk Difference (95% CI)	Certainty	Risk Difference (95% CI)	Certainty
Saglimbene 2020 [22]	SMD 0.22 SD (−0.03 to 0.48)	LOW	**RR 0.3** **(0.09 to 0.98)**	VERY LOW	MD 2.20 (−17.63 to 22.03)	VERY LOW	MD −0.16 (−0.48 to 0.15)	LOW	-	-
Hu 2017 [43]	SMD 0.14 SD (−0.13 to 0.42)	LOW	**RR 0.49** **(0.24 to 0.99)**	MODERATE	-	-	**SMD −0.31 SD** **(−0.53 to −0.10)**	LOW	SMD 0.22 SD (−0.40 to 0.84)	LOW

The bold of the words in the context means a statistical significance. eGFR: estimated glomerular filtration rate; ESKD: end-stage kidney diseases; SCr: serum creatinine; CCr: creatinine clearance; CI: Confidence interval; MD: Mean difference; SMD: Standardized mean difference; RR: Risk ratio.

**Table 6:** Summary of clinical important outcomes and certainties on the renal protection effect of biotics compared to placebo.

Author Year/Clinical Important Outcomes	Serum Urea		BUN		SCr	
	Risk Difference (95% CI)	Certainty	Risk Difference (95% CI)	Certainty	Risk Difference (95% CI)	Certainty
McFarlane 2019 [25]	**MD −2.12 ^a^** **(−3.86 to −0.37)**	LOW	-	-	-	-
Tao 2019 [47]	**MD −30.01 ^b^** **(−56.78 to −3.25)**	MODERATE	-	-	-	-
Jia 2018 [46]	-	-	MD −5.78 (−21.42 to 9.86)	VERY LOW	MD 0.10 (−0.11 to 0.31)	VERY LOW
Pisano 2018 [23]	SMD −0.20 SD (−0.41 to 0.01)	VERY LOW	-	-	MD −0.02 (−0.09 to 0.05)	VERY LOW

The bold of the words in the context means a statistical significance. ^a^: the unit is mmol/L; ^b^: the unit is mg/dL, conversion to SI unit is MD −5.00 (−9.45 to −0.54); BUN: blood urea nitrogen, SCr: serum creatinine; CI: Confidence interval; MD: Mean difference; SMD: Standardized mean difference.

## Supplementary Materials

The following are available online at https://www.mdpi.com/2072-6643/13/2/469/s1. Table S1: PRISMA checklist of the systematic review.

## Abbreviations

BUN	blood urea nitrogen
CCr	creatinine clearance
CI	confidence interval
CKD	chronic kidney disease
CoQ10	coenzyme Q10
CRP	C-reactive protein
DN	diabetic nephropathy
eGFR	estimated glomerular filtration rate
ESKD	end stage kidney disease
ESRD	end-stage renal disease
GRADE	Grading of Recommendations Assessment, Development, and Evaluation
hs-CRP	high-sensitivity C-reactive protein
IL-6	interleukin 6
IS	indoxyl sulphate
MD	mean difference
MDA	malondialdehyde
OIS	optimal information size
PCS	p-cresyl sulphate
PRISMA	Preferred Reporting Items for Systematic Reviews and Meta-Analyses
PUFA	polyunsaturated fatty acid
RCTs	randomized-controlled trials
RoB	risk of bias
RR	risk ratio
SCr	serum creatinine
SMD	standardized mean difference
SR	systematic review
T1DN	type 1 diabetic nephtopathy
T2DN	type 2 diabetic nephtopathy
TNF-α	tumor necrosis factor-alpha
UACR	urine albumin creatinine ratio
UAER	urinary albumin excretion rate
UPCR	urine protein creatinine ratio
